# Analysis of *Bordetella pertussis* clinical isolates circulating in European countries during the period 1998–2012

**DOI:** 10.1007/s10096-014-2297-2

**Published:** 2014-12-20

**Authors:** M. van Gent, C. J. Heuvelman, H. G. van der Heide, H. O. Hallander, A. Advani, N. Guiso, C. H. Wirsing von Kőnig, D. F. Vestrheim, T. Dalby, N. K. Fry, D. Pierard, L. Detemmerman, J. Zavadilova, K. Fabianova, C. Logan, A. Habington, M. Byrne, A. Lutyńska, E. Mosiej, C. Pelaz, K. Gröndahl-Yli-Hannuksela, A. M. Barkoff, J. Mertsola, A. Economopoulou, Q. He, F. R. Mooi

**Affiliations:** 1 Centre for Infectious Disease Control (CIb), National Institute for Public Health and the Environment (RIVM), P.O. Box 1, 3720 BA Bilthoven, The Netherlands; 2 The Swedish National Institute of Public Health, Solna, Sweden; 3 Molecular Prevention and Therapy of Human Diseases, Institut Pasteur, Paris, France; 4 Labor: Medizin Krefeld MVZ, Helios Klinikum, Krefeld, Germany; 5 Department of Bacteriology and Immunology, Norwegian Institute of Public Health, Oslo, Norway; 6 Department of Microbiology and Infection Control, Statens Serum Institut, Copenhagen, Denmark; 7 Respiratory and Vaccine Preventable Bacteria Reference Unit, Public Health England—Microbiology References Services, Colindale, United Kingdom; 8 National Reference Center for Pertussis, Department of Microbiology and Infection Control, Universitair Ziekenhuis Brussel, Brussels, Belgium; 9 National Reference Laboratory for Pertussis and Diphtheria, National Institute of Public Health, Prague, Czech Republic; 10 Department of Infectious Diseases Epidemiology, National Institute of Public Health, Prague, Czech Republic; 11 Microbiology Department, Our Lady’s Children’s Hospital, Dublin, Ireland; 12 Department of Sera and Vaccines Evaluation, National Institute of Public Health, National Institute of Hygiene, Warsaw, Poland; 13 Centro Nacional de Microbiología, Instituto de Salud Carlos III, Majadahonda, Madrid Spain; 14 Department of Infectious Disease Surveillance and Control, National Institute for Health and Welfare, Turku, Finland; 15 Department of Pediatrics, Turku University Hospital, Turku, Finland; 16 European Centre for Disease Control and Prevention (ECDC), Stockholm, Sweden

## Abstract

Despite more than 50 years of vaccination, pertussis is still an endemic disease, with regular epidemic outbreaks. With the exception of Poland, European countries have replaced whole-cell vaccines (WCVs) by acellular vaccines (ACVs) in the 1990s. Worldwide, antigenic divergence in vaccine antigens has been found between vaccine strains and circulating strains. In this work, 466 *Bordetella pertussis* isolates collected in the period 1998–2012 from 13 European countries were characterised by multi-locus antigen sequence typing (MAST) of the pertussis toxin promoter (*ptxP*) and of the genes coding for proteins used in the ACVs: pertussis toxin (Ptx), pertactin (Prn), type 2 fimbriae (Fim2) and type 3 fimbriae (Fim3). Isolates were further characterised by fimbrial serotyping, multi-locus variable-number tandem repeat analysis (MLVA) and pulsed-field gel electrophoresis (PFGE). The results showed a very similar *B. pertussis* population for 12 countries using ACVs, while Poland, which uses a WCV, was quite distinct, suggesting that ACVs and WCVs select for different *B. pertussis* populations. This study forms a baseline for future studies on the effect of vaccination programmes on *B. pertussis* populations.

**Electronic supplementary material:**

The online version of this article (doi:10.1007/s10096-014-2297-2) contains supplementary material, which is available to authorized users.

## Introduction

Pertussis, or whooping cough, is a highly contagious human infection of the upper respiratory tract caused by the Gram-negative bacterium *Bordetella pertussis* [1]. Despite the introduction of vaccination against pertussis more than 50 years ago, the disease is still a public health problem worldwide, with epidemic outbreaks occurring every 3–5 years. In the last 10 years, a resurgence of pertussis was observed in countries with highly vaccinated populations, including Australia [2], Norway [3], Poland [4], the Netherlands [5], the United Kingdom [6] and the United States [7]. One of the hallmarks of the pertussis resurgence is the shift in prevalence from young infants to older persons with waning vaccine-induced immunity [8, 9]. Several causes have been proposed for the resurgence of pertussis, including improved diagnosis, increased awareness [10, 11], decreased vaccine efficacy, waning immunity and pathogen adaptation [12, 13]. The contribution of these factors to the resurgence of pertussis may differ between countries.

European countries use different vaccines and vaccination schedules for pertussis (Table 1 and http://vaccine-schedule.ecdc.europa.eu/Pages/Scheduler.aspx). Partly due to the side effects of the whole-cell vaccines (WCVs), all European countries, except Poland, have replaced WCVs by acellular vaccines (ACVs) since the 1990s [14]. WCVs are composed of killed *B. pertussis* bacteria, while ACVs consist of 1–5 purified *B. pertussis* proteins: pertussis toxin (Ptx), pertactin (Prn), filamentous haemagglutinin (FHA), type 2 fimbriae (Fim2) and type 3 fimbriae (Fim3). Worldwide, variation in these vaccine antigens have been found between strains used for the production of the vaccines and isolates that are circulating in those countries where these vaccines have been used extensively [2, 15–25].

**Table 1:** Pertussis vaccines currently used in European countries*

Country	Vaccine
Belgium	ACV3
Czech Republic	ACV3 or ACV5
Denmark	ACV1
Finland	ACV2 or ACV3
France	ACV2, ACV3 or ACV5
Germany	ACV2 or ACV3
Ireland	ACV3
Norway	ACV3
Poland	WCV
Spain	ACV3
Sweden	ACV2 or ACV3
The Netherlands	ACV3
United Kingdom	ACV3 or ACV5

*Vaccine compositions: ACV1: Ptx; ACV2: Ptx and FHA; ACV3: Ptx, FHA and Prn; ACV5: Ptx, FHA, Prn, Fim2 and Fim3

Besides antigenic variation, recently, *B. pertussis* isolates have emerged which produce higher levels of a number of virulence factors in vitro, including Ptx [5, 26]. These isolates carry a new allele for the Ptx promoter, *ptxP3*, and have replaced the resident *ptxP1* isolates in many countries. The emergence of *ptxP3* isolates is associated with the increase in pertussis notifications in the Netherlands since 1996 [5]. Nowadays, *ptxP3* isolates are found worldwide [20–22, 25, 27–30]. Moreover, recently, *B. pertussis* isolates have been observed that do not express one or more vaccine components, in particular, Prn [7, 31–39].

Monitoring changes in the European *B. pertussis* populations and studying the impact of these changes on the disease burden are important in order to establish the most effective pertussis vaccines and vaccination strategies. To study these changes, a European network, designated EUpertstrain, was created in 2001, with the main aim of collecting and typing of European *B. pertussis* isolates. The number of participating countries increased from five in 2001 to nine in 2013 [40–43]. Members of the EUpertstrain group collected and typed isolates on a regular basis between 2001 and 2013. Typing involved multi-locus antigen sequence typing (MAST), fimbrial serotyping, multi-locus variable-number tandem repeat analysis (MLVA) and pulsed-field gel electrophoresis (PFGE). However, direct comparison of these isolates collected during the study period was only performed by PFGE analyses [41–43].

This present study is part of a European Centre for Disease Control and Prevention (ECDC)-funded network, EUpert-labnet, which focuses on the laboratory surveillance of whooping cough in EU Member States and European Economic Area (EEA) countries. In this study, we present the typing results of isolates collected from 13 countries (Belgium, the Czech Republic, Denmark, Finland, France, Germany, Ireland, Norway, Poland, Spain, Sweden, the Netherlands and the United Kingdom) between 2000 and 2012. Further, the typing of previous collections was extended and the results were combined to give an overview of *B. pertussis* isolates circulating in European countries between 1998 and 2012.

## Materials and methods

### *B. pertussis* isolates

*B. pertussis* isolates were grown on Bordet–Gengou (BG) agar with 15 % sheep blood and incubated for 3 to 4 days at 35 °C. In total, 466 *B. pertussis* isolates were included in this study (Supplementary Table S1). Isolates collected between 1998 and 2009 were previously analysed by PFGE [41–43]. Here, we extended these analyses by typing more isolates from this period. Further, we present novel data from five EU countries; Belgium (*n* = 20, isolated in 2000–2012), the Czech Republic (*n* = 20, isolated in 2008–2012), Ireland (*n* = 20, isolated in 2003–2012), Poland (*n* = 20, isolated in 2000–2012) and Spain (*n* = 12, isolated in 2004–2012).

### Multi-locus antigen sequence typing (MAST)

Polymorphisms in the genes for proteins used in the ACVs (PtxA, Prn, Fim2 and Fim3) were analysed as described previously [5, 15, 44, 45]. The pertussis toxin promoter, *ptxP*, was also included, as previous studies have shown that the *ptxP3* allele is an important characteristic of successful isolates [5, 20–22, 25, 27–30]. For DNA isolation, bacterial cells were lysed in Tris-EDTA buffer (Sigma-Aldrich, Zwijndrecht, the Netherlands, 1.0 M Tris–HCl, containing 0.1 M EDTA, 100× concentrated) at 95 °C for 5 min, centrifuged briefly and used in a polymerase chain reaction (PCR) assay.

### Serotyping

A bacterial suspension was mixed on a glass slide with monoclonal or polyclonal antibodies against Fim2 or Fim3 (National Institute for Biological Standards and Control (NIBSC), South Mimms, UK). Agglutination was determined after a maximum of 30 s to avoid false-positive agglutination [44]. Bacterial suspensions were mixed with a physiological salt solution to determine auto-agglutination.

### Multi-locus variable-number tandem repeat analysis (MLVA)

For MLVA, the variable number of tandem repeats in six loci (VNTR1, VNTR3a, VNTR3b, VNTR4, VNTR5 and VNTR6) was determined as described previously [23, 46].

### Pulsed-field gel electrophoresis (PFGE)

All EUpertstrain isolates were previously analysed by PFGE at the Swedish Institute for Communicable Disease Control (SMI) [43]. PFGE of isolates from Belgium, Ireland and Poland was performed by the respective countries, whereas PFGE of isolates from the Czech Republic and Spain was performed at the Finnish National Institute for Health and Welfare (THL). Gel images of all isolates from the five countries were also analysed at the Finnish THL. The PFGE protocol, reference strains used and profile analysis were the same as those previously described [43]. The profiles were analysed by using BioNumerics software version 4.61 (Applied Maths, Sint-Martens-Latem, Belgium). The Swedish nomenclature was used and based on cluster analysis with the group method with 1 % band tolerance and 1 % optimisation settings. The resulting profiles were designated BpSR1, BpSR2, BpSR3 etc. for those isolates with profiles first detected in Sweden. Isolates first identified in a country other than Sweden, such as Finland, were designated BpFINR1, BpFINR2 etc.

## Results

### The EUpertstrain and EUpert-labnet collections

One of the aims of the EUpertstrain network is to collect isolates from different European countries in order to assess the emergence and spread of new variants of *B. pertussis*. A secondary aim is to determine the effect, if any, of different vaccination strategies on the pertussis burden and the emergence of new variants. Analyses of the three EUpertstrain collections isolated between 1998 and 2009 by PFGE and analysis of the EUpert I collection isolated between 1998 and 2001 by MLVA have already been published [40–43].

Here, we extend this work to the EUpert-labnet collection by including five more EU countries: Belgium, the Czech Republic, Ireland, Poland and Spain (Table 2). Further, additional typing was performed on previously collected isolates. The old and new data were integrated to give an overview of (country-specific) changes of the *B. pertussis* populations in the participating European countries. Isolates collected between 1998 and 2012 were aggregated in three periods to obtain a comparable number of isolates per year; 1998–2001 (*n* = 106), 2002–2006 (*n* = 165) and 2007–2012 (*n* = 195). As PCR is replacing culture for pertussis diagnosis, obtaining sufficient strains for population studies has become difficult. The number of participating countries for the three periods was 7, 11 and 12, respectively (Table 2). For Finland, France, the Netherlands and Sweden, 17 to 23 isolates were available for each period. In contrast, for Norway and the Czech Republic, isolates were only available for period 2007–2012. Table 1 shows the pertussis vaccines currently used in European countries.

**Table 2:** Number of *Bordetella pertussis* clinical isolates used in this study

Country	1998–2001	2002–2006	2007–2012	Total
Belgium	2	8	10	20
Czech Republic	0	0	20	20
Denmark	0	20	23	43
Finland	20	20	17	57
France	20	20	20	60
Germany	17	18	0	35
Ireland	0	5	15	20
Norway	0	0	20	20
Poland	11	7	2	20
Spain	0	2	10	12
Sweden	17	20	20	57
The Netherlands	19	23	20	62
United Kingdom	0	22	18	40
Total	106	165	195	466

### Changes in allele frequencies

We investigated changes in frequencies of the alleles for four proteins used in ACVs: *ptxA*, *prn*, *fim2* and *fim3*. The pertussis toxin promoter, *ptxP*, was also included, as several previous studies have shown that the *ptxP3* allele is an important characteristic of recent clinical isolates [5, 20, 28]. Isolates were also serotyped to assess expression of the *fim2* and *fim3* genes. All allelic variants discussed here are associated with changes in protein structure. An overview of allelic and protein variants is provided in previously published reviews [12, 47].

For our analysis, we included data only if at least five isolates were available in a particular period. In general, Poland was found to be distinct, while the remaining 12 countries showed minor differences in their *B. pertussis* populations. Therefore, except for the *ptxA* alleles and serotypes, we limited the comparisons to Poland and pooled the remaining 12 countries. All data are represented in Supplementary Table S1.

#### *ptxA* alleles

The ACVs currently used in the European countries contain *ptxA2* and *ptxA4* [12] and the Polish WCV contains only *ptxA2* [48]. Essentially, no differences were found between the 13 countries with respect to *ptxA* alleles. The non-vaccine type allele *ptxA1* was identified in 428 out of the 429 isolates analysed. One isolate, isolated in Sweden in 1998, harboured the *ptxA2* allele.

#### *prn* alleles

**Fig. 1:**
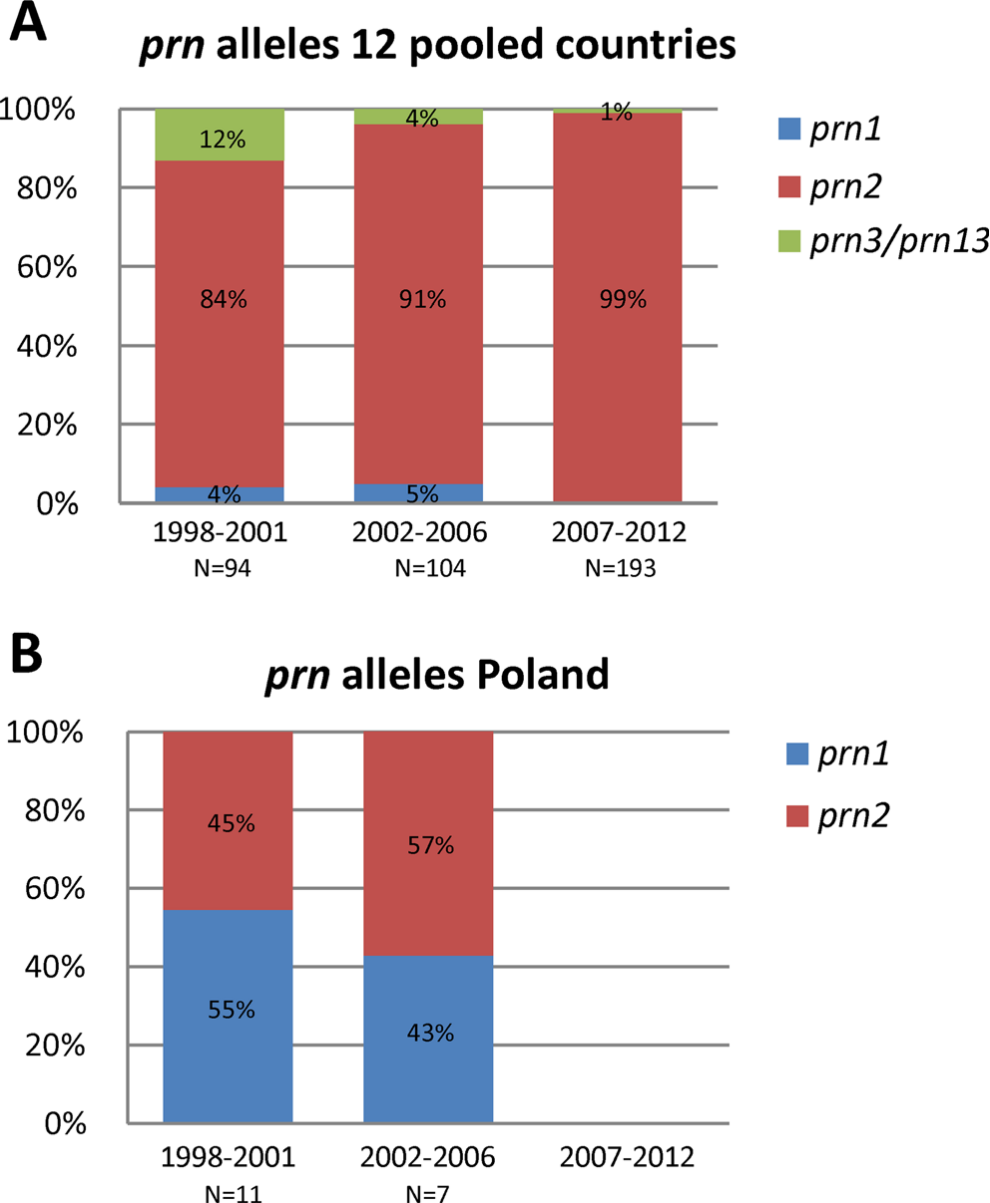
Frequencies of the *prn* alleles in the period 1998–2012. Isolates were aggregated in three periods, 1998–2001, 2002–2006 and 2007–2012. **a** Allele frequencies in the 12 pooled countries: Belgium, the Czech Republic, Denmark, Finland, France, Germany, Ireland, Norway, Spain, Sweden, the Netherlands and the United Kingdom. **b** Allele frequencies in Poland. Due to the limited availability of Polish isolates in the period 2007–2012 (*n* = 2), no data are included for this period. The percentages and number of strains analysed in the different periods are indicated

The ACVs currently used in the European countries contain *prn1* and *prn7* [12] and the Polish WCV contains only *prn1* [48]. In the group of 12 countries, four *prn* alleles were observed in this study, *prn1*, *prn2*, *prn3* and *prn13*. The *prn13* allele was detected once in Sweden during the period 1998–2001. The *prn3* allele was found in all three periods but decreased in frequency from 10 % in 1998–2001 to 4 % in 2002–2006 and to 1 % in 2007–2012. The two minor *prn* alleles *prn13* and *prn3* were combined into one group in Fig. 1a. In the 12 countries, *prn2* predominated in 1998–2001 with a frequency of 84 %, increasing to 91 % in 2002–2006 and to 99 % 2007–2012 (Fig. 1a). In Poland, the frequencies of *prn1* in the periods 1998–2001 and 2002–2006 were, respectively, 55 and 43 % (Fig. 1b). Two Polish isolates were available in 2007–2012, both containing *prn1*.

#### *ptxP* alleles

**Fig. 2:**
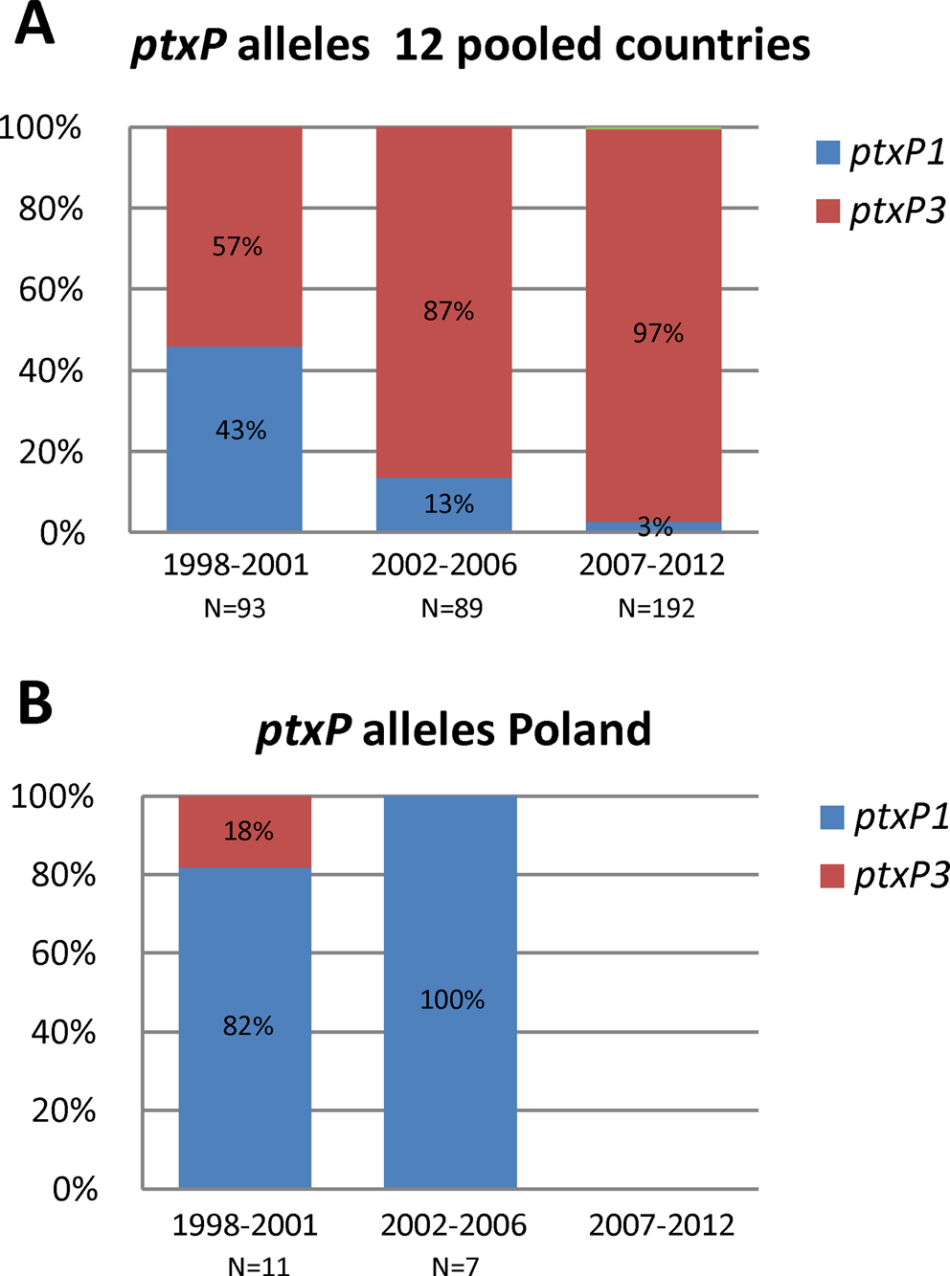
Frequencies of the *ptxP* alleles in the period 1998–2012. See caption of Fig. 1 for further details

Three *ptxP* alleles were found in this study, *ptxP1*, *ptxP3* and *ptxP20*. *ptxP20* was found once in the Czech Republic in 2007–2012 and not included in Fig. 2. An increasing prevalence of *ptxP3* isolates was observed in the group of 12 countries from 57 % in 1998–2001 to 87 % in 2002–2006 and to 97 % in 2007–2012 (Fig. 2a). In Poland, a distinct trend was found. *ptxP1* predominated in both periods 1998–2001 and 2002–2006, with frequencies of 82 and 100 %, respectively (Fig. 2b). Only two Polish isolates were available in 2007–2012, both containing *ptxP1*.

#### *fim2* alleles

**Fig. 3:**
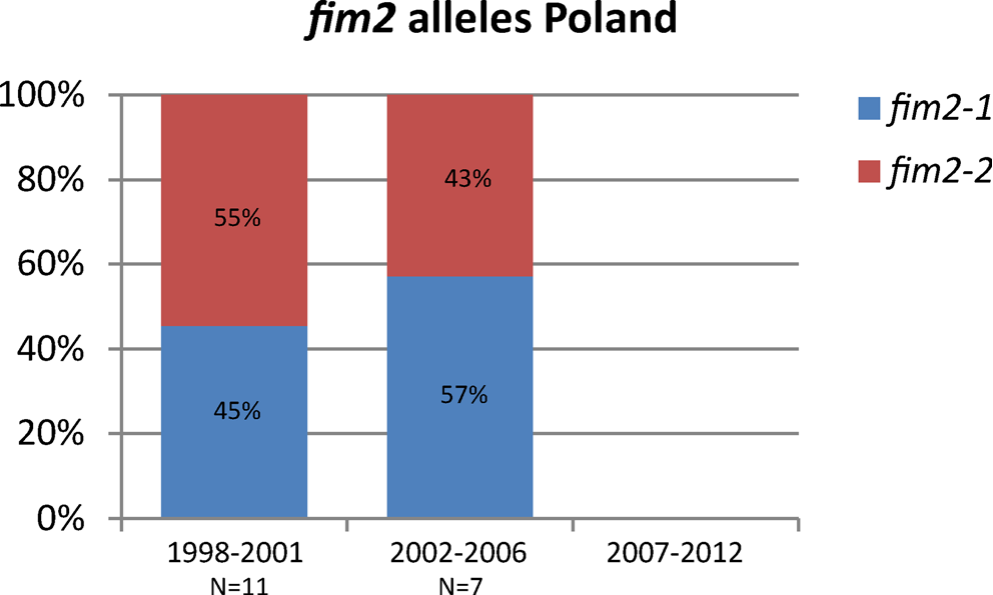
Frequencies of the *fim2* alleles in the period 1998–2006 in Poland. Two periods are indicated, 1998–2001 and 2002–2006. Due to the limited availability of Polish isolates in 2007–2012 (*n* = 2), no data are included for this period. In the remaining 12 countries, only *fim2*-*1* was observed, with the exception of Belgium, where one *fim2*-*2* isolate was identified

Two *fim2* alleles have been found worldwide, *fim2*-*1* and *fim2*-*2* [47]. Only *fim2*-*1* is present in the currently used ACVs [12] and the Polish WCV [48]. In this collection, *fim2*-*2* isolates were only detected in Poland and Belgium, while the remaining isolates harboured the *fim2*-*1* allele. In Belgium, one *fim2*-*2* isolate was observed in the period 2002–2006. In Poland, the *fim2*-*2* frequencies were 55 % in 1998–2001 and 43 % in 2002–2006 (Fig. 3). Only two Polish isolates were available for the period 2007–2012: one isolate harboured the *fim2*-*1* allele and one harboured the *fim2*-*2* allele; therefore, the prevalences for the period 2007–2012 were not included in the figure.

#### *fim3* alleles

**Fig. 4:**
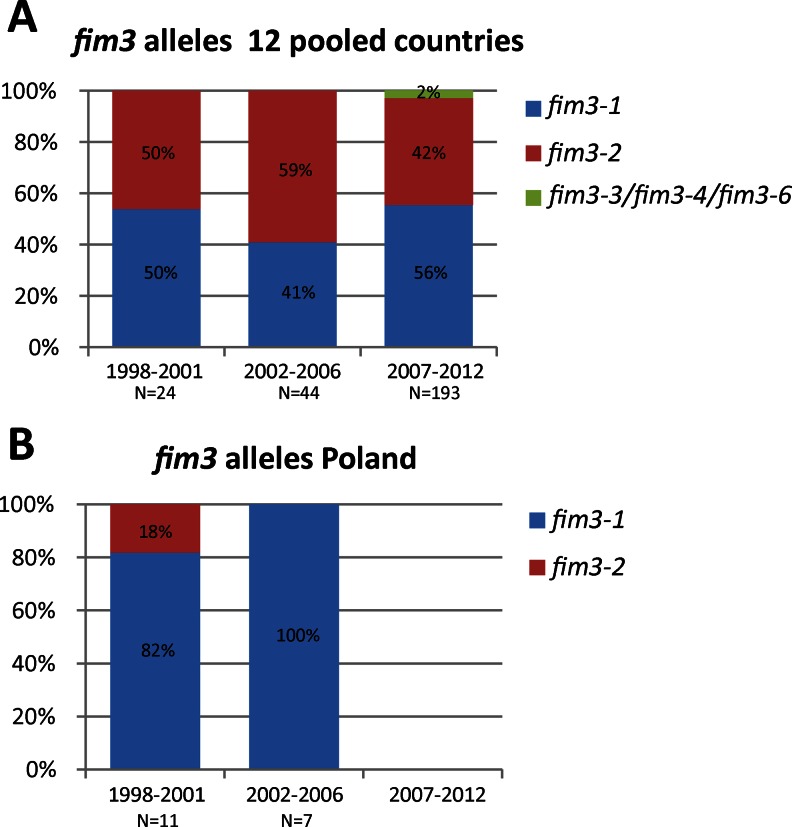
Frequencies of the *fim3* alleles in the period 1998–2012. See caption of Fig. 1 for further details

Five *fim3* alleles were found in this study, *fim3*-*1* (found in ACVs [12]) and the Polish WCV [48]), *fim3*-*2*, *fim3*-*3*, *fim3*-*4* and *fim3*-*6*. Two *fim3*-*3* isolates were found, one in the Netherlands (in the period 2007–2012) and one in Denmark (in the period 2007–2012). A *fim3*-*4* isolate was found in France (in the period 2007–2012) and a *fim3*-*6* isolate was found in Belgium (in the period 2007–2012). The three minor *fim3* alleles, *fim3*-*3*, *fim3*-*4* and *fim3*-*6*, were combined into one group in Fig. 4a. In the group of 12 countries, *fim3*-*1* was found with frequencies of 50, 41 and 56 % in the periods 1998–2001, 2002–2006 and 2007–2012, respectively (Fig. 4). In Poland, *fim3*-*1* predominated in the period 1998–2001 (frequency 82 %) and increased in frequency to 100 % in the period 2002–2006 (Fig. 4b). Only two Polish isolates were available for the period 2007–2012, both containing *fim3*-*1*.

### Fimbrial serotyping

**Fig. 5:**
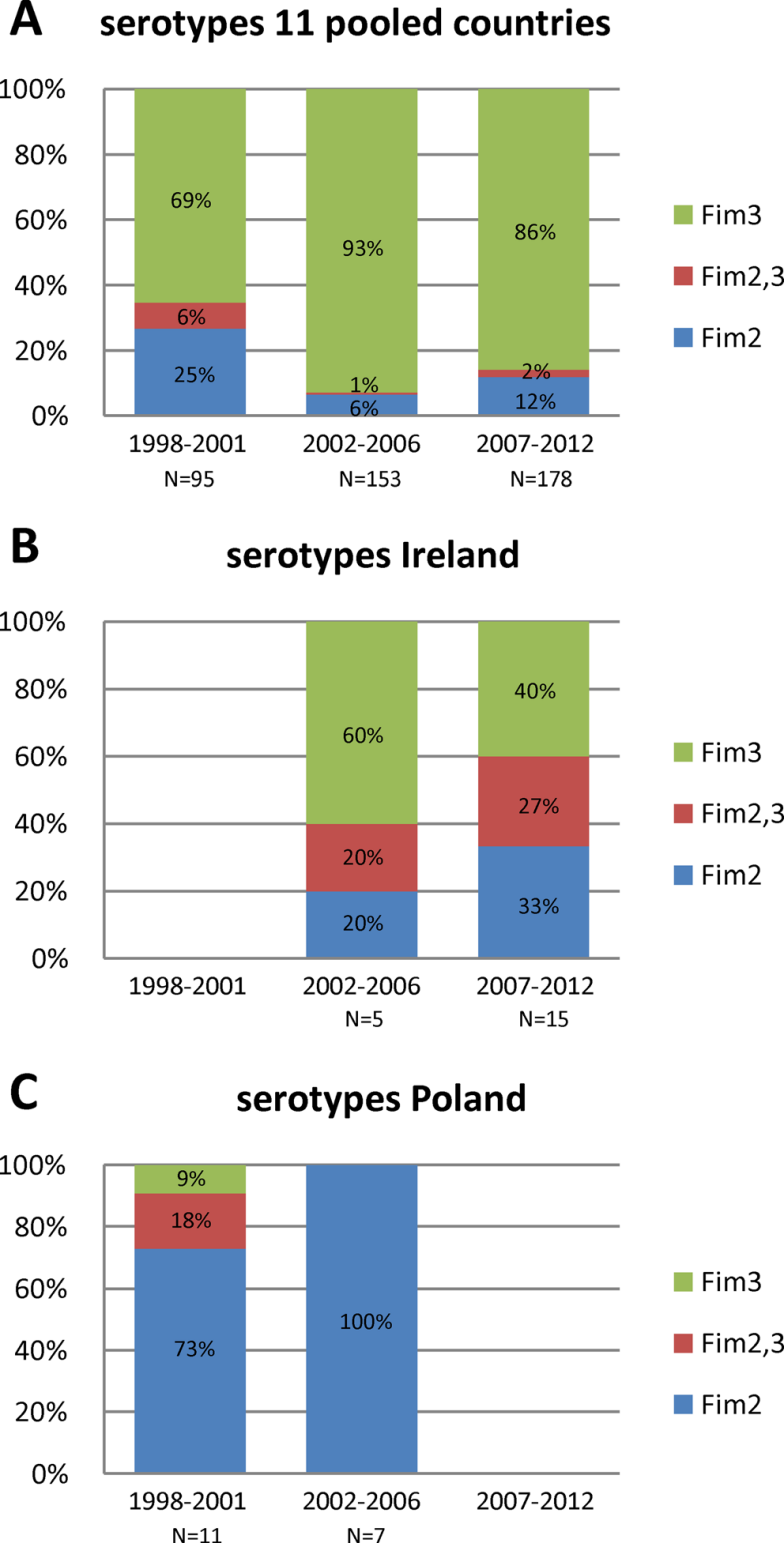
Frequencies of the serotypes in the period 1998–2012. **a** Serotype frequencies of the 11 pooled countries; Belgium, the Czech Republic, Denmark, Finland, France, Germany, Norway, Spain, Sweden, the Netherlands and the United Kingdom. **b** Serotype frequencies in Ireland. **c** Serotype frequencies in Poland. See caption of Fig. 1 for details

*B. pertussis* produces two serologically distinct fimbriae, designated serotype 2 (Fim2) and serotype 3 fimbriae (Fim3). A *B. pertussis* isolate may produce a single serotype or both serotypes. Therefore, three combinations are possible: Fim2, Fim3 and Fim2,3. Ireland and Poland were distinct with respect to fimbrial serotypes, whereas minor differences were found between the remaining 11 countries. Therefore, Ireland and Poland were treated separately, while the data of the remaining 11 countries were pooled. Fim3 predominated in the group of 11 countries with frequencies of 69 % in 1998–2001, 93 % in 2002–2006 and 86 % in 2007–2012 (Fig. 5a). In Ireland, lower frequencies of Fim3 were found: 60 % in 2002–2006 and 40 % in 2007–2012. No Irish isolates were available for the period 1998–2001 (Fig. 5b). In Poland, Fim2 predominated in 1998–2001 and 2006–2006 (frequencies of 73 and 100 %, respectively) (Fig. 5c). Only two Polish isolates were available in 2007–2012, both of which were Fim2. The Polish WCV is derived from strains which produce both Fim2 and Fim3 [49]. One strain, isolated in Spain in 2010, was found to be auto-agglutinable (data not shown).

### Multi-locus variable-number tandem repeat analysis (MLVA)

**Fig. 6:**
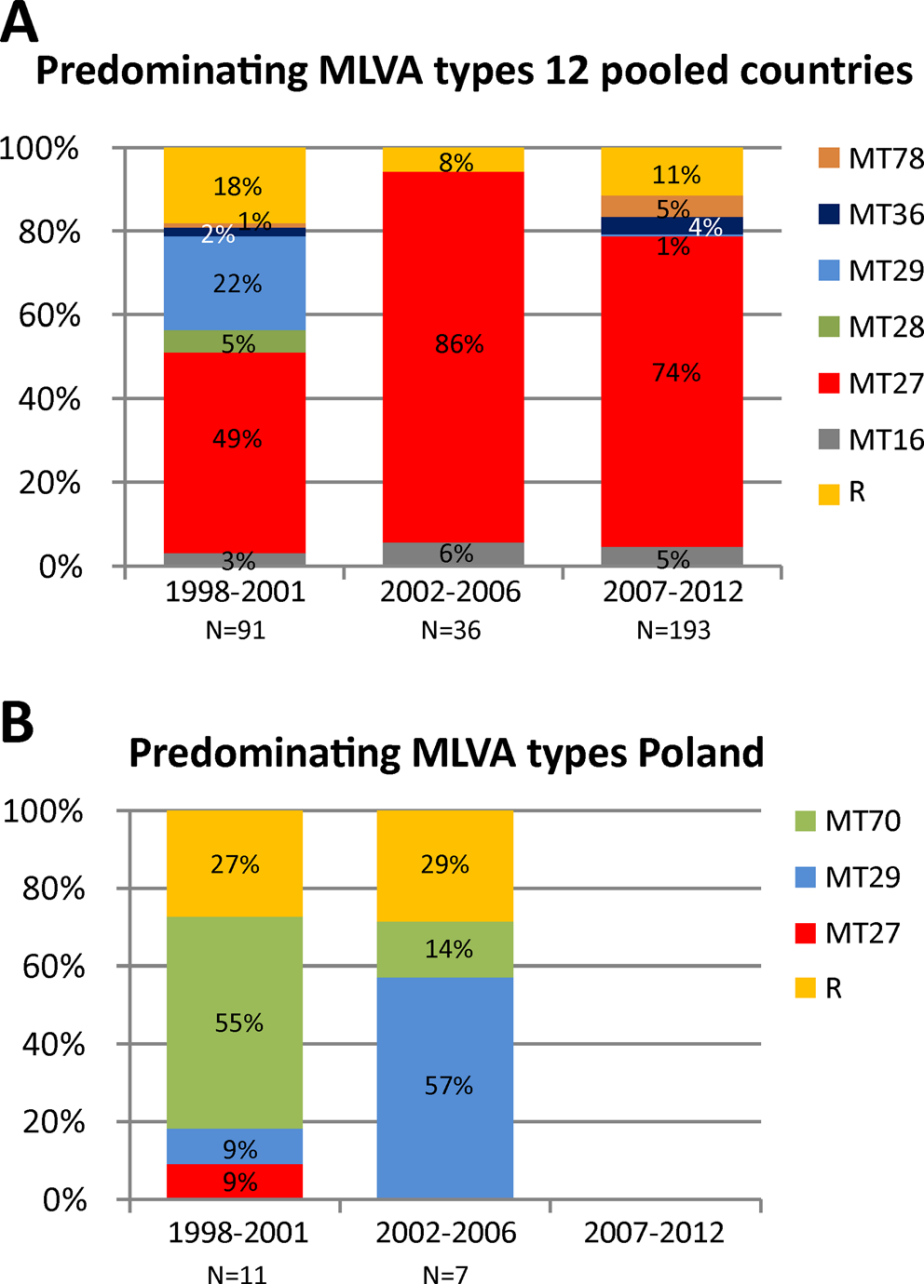
Frequencies of the predominating multi-locus variable-number tandem repeat analysis (MLVA) types in the period 1998–2012. See caption of Fig. 1 for further details

Thirty-four MLVA types (MTs) were found in the 13 countries. MTs that were found less than five times in the group of 12 countries or in Poland were combined and designated as group R in Fig. 6a, b. MT27 predominated in the group of 12 countries (frequencies from 54 to 100 % per country) but was found once in Poland (in 1994–2001). MT27 predominated in 1998–2001, with a frequency of 49 %, which increased to 86 % in 2002–2006 and decreased to 74 % in 2007–2012 (Fig. 6a). MT29 predominated only in 1998–2001, with a frequency of 22 %, which decreased to 0 % in 2002–2006 and increased slightly to 1 % in 2007–2012. MT78 predominated in Finland in 2007–2012 (data not shown) (frequency 53 %). MT78 was found once in Norway (in the period 2007–2012) and once in Germany (in the period 1998–2001). In Poland, MT70 predominated, with frequencies of 55 % in 1998–2001 and 14 % in 2002–2006 (Fig. 6b). Besides Poland, MT70 has been found once in Belgium in the period 2002–2006. During the period 2002–2006, MT29 predominated in Poland, with a frequency of 57 %.

### Pulsed-field gel electrophoresis (PFGE)

**Fig. 7:**
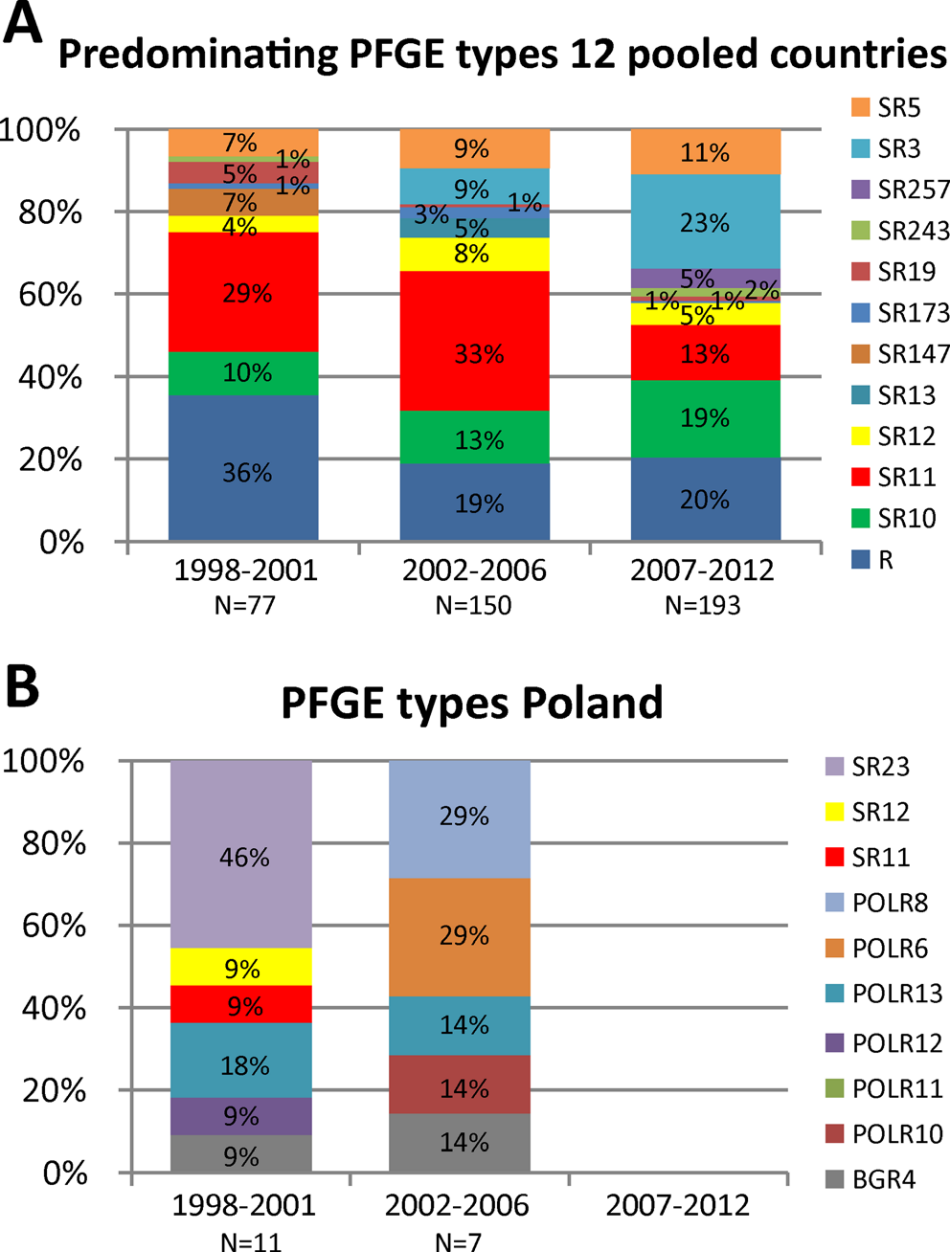
Frequencies of the predominating pulsed-field gel electrophoresis (PFGE) types in the period 1998–2012. See caption of Fig. 1 for further details

Eighty-seven PFGE types were found in the 13 countries. PFGE types that were found less than five times in the group of 12 countries were combined and designated as group R in Fig. 7a. In the group of 12 countries, increasing frequencies were observed for BpSR3 (0 % in 1998–2001 to 23 % in 2007–2012) and BpSR10 (10 % in 1998–2001 to 19 % in 2007–2012). Decreasing trends have been found for BpSR11 (29 % in 1998–2001 to 13 % in 2007–2012). No differences were found for the frequencies of BpSR5 (7 % in 1998–2001, 9 % in 2002–2006 and 11 % in 2007–2012) and BpSR12 (4 % in 1998–2001, 8 % in 2002–2006 and 5 % in 2007–2012) (Fig. 7a). In Poland, BpSR23 predominated in the period 1998–2001 (frequency 46 %). The predominating PFGE type in Europe, BpSR11, was found once in Poland, in the period 1998–2001 (Fig. 7b).

## Discussion

In this study, we analysed *B. pertussis* isolates from 13 European countries during the period 1998–2012. In general, our comparison showed that 12 countries had very similar *B. pertussis* populations (Belgium, the Czech Republic, Denmark, Finland, France, Germany, Ireland, the Netherlands, Norway, Spain, Sweden and the United Kingdom), while Poland was quite distinct. As noted previously, in all countries, circulating isolates were distinct from vaccine strains with respect to one or more of the investigated antigens [2, 15–25].

The *ptxA* gene was found to be highly monomorphic and, with one exception, all circulating isolates carried the non-vaccine type allele *ptxA1*. Three *ptxP* alleles were observed in this collection of isolates, *ptxP1*, *ptxP3* and *ptxP20*. A notable observation was the low percentage of *ptxP3* isolates in Poland (10 %, *n* = 20) compared to the other countries (average 72 %, range 37–100 %). Interestingly, the two Polish *ptxP3* isolates were found in the first period, 1998–2001, but not in the later periods. Possibly, *ptxP3* isolates do not have a fitness advantage in Poland, in contrast to the other 12 countries. Further, although the non-vaccine type allele *prn2* was found at frequencies of 45 and 57 % in the periods 1998–2001 and 2002–2006 in Poland, respectively, these percentages were lower than those observed in the remaining 12 countries (average 83 %, range 37–100 %). Comparing the clinical outcome between *ptxP1*- and *ptxP3*-infected individuals, especially in Poland, would be useful.

In Poland, two *fim2* alleles circulated in approximately equal frequencies, *fim2*-*1* (the vaccine type in Poland) and *fim2*-*2*. In contrast, in the remaining countries, only *fim2*-*1* was observed, with the exception of Belgium, where a single *fim2*-*2* isolate was identified. The Belgian *fim2*-*2* isolate was linked to the same MLVA and PFGE type (MT70 and BpBGR4, respectively) as 30 % of the Polish *fim2*-*2* isolates, suggesting that the *fim2*-*2* isolate was introduced in Belgium from Poland. The *fim2*-*2* allele was also observed in the pre-vaccination era in the Netherlands (57 %, *n* = 7), while all isolates from 1965 or later harboured *fim2*-*1* [45]. More recently, *fim2*-*2* isolates were observed in Moscow (89 % in the period 1990–2004) and Australia (36 % in the period 1998–2008) [50, 51]. In both Poland and Australia, a significant percentage of the *fim2*-*2* isolates were linked to MT70 (80 and 88 %, respectively), possibly suggesting a common origin. However, independent genesis and fixation of the *fim2*-*2* single nucleotide polymorphism (SNP) by different *B. pertussis* lineages may also be possible. The latter would suggest that the mutation has a significant effect on fitness.

The limited distribution of *fim2*-*2* isolates in Europe compared to Moscow and Poland could be due to geographic isolation, as travelling to Poland and/or Moscow from Western Europe was restricted until the late 1980s. However, in the Czech Republic, which was also located in the former Eastern Europe, no *fim2*-*2* isolates have been observed. Another cause for the observation that the distribution of *fim2*-*2* isolates was limited to Poland and Russia could be the use of the pertussis vaccines, as in Poland and Moscow, a WCV is still used, while an ACV has already been introduced in the Czech Republic in 2007.

The Polish and Irish populations showed higher Fim2 frequencies compared to the other 11 countries (80 and 30 %, respectively). An association between Fim2 and the prevalence of *fim2*-*2* isolates has been found previously in the United Kingdom, where isolates collected between 1920 and 2002 were studied [52]. All isolates with the *fim2*-*2* allele (*n* = 20) were Fim2. This was also observed in Poland, where 90 % of the *fim2*-*2* isolates were Fim2. However, no *fim2*-*2* alleles have been found in Ireland in this study.

MT27 is the predominating MT in Europe. Previous European studies showed a linkage between *ptxP3* and MT27 [17, 18, 46]. Indeed, 97 % of MT27 isolates in this study harboured the *ptxP3* allele. MT27 was also the predominating MT in countries outside Europe, including Australia [2], Japan [25] and the United States [22]. One of the two Polish *ptxP3* isolates was also typed as MT27.

The Fim and Prn types of Danish isolates were similar to the isolates found in other countries, except Poland. Since the Danish vaccine contains only Ptx, the changes to the Fim and Prn types in Danish isolates may be the result of overflow from neighbouring countries, rather than selective pressure from the Ptx vaccine.

Five predominant PFGE types have been found in Europe, BpSR3, BpSR5, BpSR10, BpSR11 and BpSR12 [43]. These PFGE types were also predominant in at least one Canadian province, where 317 out of 434 (73 %) isolates collected between 1998 and 2006 belonged to the PFGE types BpSR5, BpSR11 and BpSR12 [24]. However, no isolate with PFGE types BpSR3 and BpSR10 have been found in Canada so far, suggesting some geographic differences in isolates.

The present study showed minor differences in European countries which used ACVs based on MAST, fimbrial serotyping, MLVA and PFGE. However, Poland, the only country included in this study where a WCV is still used, showed a distinct population.

A distinct population compared to Europe has also been found in Serbia, where a WCV has been continued to be used since 1957 [53]. Both countries, Poland and Serbia, have a vaccination history including several changes of strain compositions since the 1960s, which could explain the distinct *B. pertussis* populations compared to the European countries where an ACV is used. The Polish and Serbian *B. pertussis* populations both show high *prn1* frequencies, 43 % in 2002–2006 and 47 % in 1985–2000, respectively [43], compared to high *prn2* frequencies (>90 %) in European countries, where ACVs have been used for many years.

The immune response induced by the two vaccines is different. WCVs induce a broad immune response, but with relatively low titres, whereas ACVs induce high titres against only a few antigens. Theoretical studies suggest that a change from a broad to a narrow immune response will favour the emergence of escape variants [54]. Indeed, several studies have shown the emergence of Prn-deficient strains in populations where ACVs are used [7, 31–39]. This is in contrast with Poland and Serbia, where a WCV has been used for many years. No Prn-deficient strains have been detected in both countries [48, 53].

This study has some limitations. Isolates were not sampled randomly and the sample size from some countries was small. However, as culture is being replaced by PCR, less and less isolates are available for typing and most members of this study sent all isolates available. Further, it was not possible to establish relationships between strain type and vaccination status, as very limited vaccination data were available. Further, FHA, also a component in some ACVs, was not included, as the large size of the gene precluded analyses. Interestingly, FHA-deficient isolates have been described [32]. In the future, targeted sequencing of genes for vaccine antigens may be replaced by whole genome sequencing, which may reveal other relevant loci to be included in MAST [51, 55]. This study was not set up to identify isolates not producing Prn in Europe, as a recent study has already addressed this issue [38]. Overall, low prevalences (<4 %) of Prn-deficient strains were found, except in Norway and France (25 % in 2007–2009 and 15 % in 2013, respectively [56]).

## Electronic supplementary material

Below is the link to the electronic supplementary material.

ESM 1 (XLSX 64 kb)
